# Airway Telomere Length in Lung Transplant Recipients

**DOI:** 10.3389/fimmu.2021.658062

**Published:** 2021-04-16

**Authors:** John A. Mackintosh, Stephanie T. Yerkovich, Maxine E. Tan, Luke Samson, Peter MA Hopkins, Daniel C. Chambers

**Affiliations:** ^1^ Queensland Lung Transplant Service, Department of Thoracic Medicine, The Prince Charles Hospital, Brisbane, QLD, Australia; ^2^ Faculty of Medicine, University of Queensland, Brisbane, QLD, Australia

**Keywords:** lung transplant, telomere, chronic lung allograft dysfunction, donor, airway

## Abstract

**Introduction:**

Chronic lung allograft dysfunction (CLAD) represents the major impediment to long term survival following lung transplantation. Donor and recipient telomere length have been shown to associate with lung transplant outcomes, including CLAD. In this study we aimed to measure the telomere lengths of bronchial and bronchiolar airway cells in lung allografts early after transplantation and to investigate associations with CLAD and all-cause mortality.

**Methods:**

This prospective, longitudinal study was performed at The Prince Charles Hospital, Australia. Airway cells were collected *via* bronchial and bronchiolar airway brushings at post-transplant bronchoscopies. The relative telomere length in airway cells was determined by quantitative PCR based on the T/S ratio. All patients were censored for CLAD and all-cause mortality in August 2020.

**Results:**

In total 231 bronchoscopies incorporating transbronchial brush and bronchial brush were performed in 120 patients. At the time of censoring, 43% and 35% of patients, respectively, had developed CLAD and had died. Airway bronchiolar and bronchial telomere lengths were strongly correlated (r=0.78, p<0.001), confirming conservation of telomere length with airway branch generation. Both the bronchiolar (r = -0.34, p<0.001) and bronchial (r = -0.31, p<0.001) telomere length decreased with age. Shorter airway telomere length was associated with older donor age and higher donor pack-year smoking history. Neither the bronchiolar nor the bronchial airway telomere length were associated with the development of CLAD (HR 0.39 (0.06-2.3), p=0.30; HR 0.66 (0.2-1.7), p=0.39, respectively) or all-cause mortality (HR 0.92 (0.2-4.5), p=0.92; HR 0.47 (0.1-1.9), p=0.28, respectively).

**Conclusions:**

In this cohort, airway telomere length was associated with donor age and smoking history but was not associated with the future development of CLAD or all-cause mortality.

## Introduction

Chronic lung allograft dysfunction (CLAD) represents the major impediment to long term survival following lung transplantation. Numerous risk factors for the development of CLAD have been described, however there is conflicting evidence on the influence of donor age. In the latest ISHLT Registry report, a small increase in the risk of CLAD in recipients of older (≥50 years) versus younger (<35 years) donors was identified and was not explained by differences in cause of death, comorbidities or smoking (1). This observation suggests that donor lung tissue retains biological ‘memory’ of age. Since passenger donor inflammatory cells are rapidly replaced by recipient cells after transplantation (2), this ‘memory’ is likely to be restricted to donor parenchymal cells (epithelium, endothelium and or stroma).

Telomeres are nucleoprotein complexes consisting of long TTAGGG repeat segments which protect chromosomes from loss of genomic material during cell replication. Telomere length is regulated by the telomerase complex of enzymes and progressive shortening occurs normally with age. Once the telomere reaches a critical threshold cellular senescence ensues. Notably, this pathway has been strongly implicated in the pathogenesis of pulmonary fibrosis (3–6). The restrictive allograft syndrome phenotype of CLAD shares many radiological and histological similarities to the idiopathic interstitial pneumonias, for which telomere shortening has been pathologically implicated (3–6). In line with the idea that shorter donor telomere length may associate with an increased risk of CLAD, shorter telomere length in donor peripheral blood mononuclear cells was observed to associate with an increased risk of CLAD in one study (7), but not with survival in another (8). In further support of an association between donor telomere length and lung transplant outcomes, both shorter epithelial telomere length (9) and markers of senescence have been identified in the obliterated airways and regions of lung fibrosis in CLAD lungs (10). Taken together, these studies suggest that donor telomere length, particularly in lung epithelium, is shorter in those who develop CLAD. However, it is not known whether this is a cause, or result, of the multiple insults which contribute to CLAD pathogenesis.

The aims of this study were to measure the telomere lengths of bronchial and bronchiolar airway cells in lung allografts early after transplantation and to investigate possible associations with CLAD and all-cause mortality. We hypothesized that shorter airway telomere length would be associated with an increased risk of CLAD.

## Methods

### Study Patients

This prospective, longitudinal study was performed at The Prince Charles Hospital with 231 bronchoscopies performed in 120 lung transplant recipients. Surveillance bronchoscopies were scheduled at 2 weeks, 6 weeks, 3 months, 6 months and 12 months post-transplant. Bronchoscopy was also indicated for patients with acute allograft dysfunction characterized by a decline (≥10%) in FEV_1_, increasing respiratory symptoms and/or new radiologic infiltrates. Transbronchial brushes were performed during the procedure as described below. Additionally, non-transplant patients undergoing a bronchoscopy for a benign, non-infective condition were recruited as healthy controls (n=10). The study was approved by The Prince Charles Hospital Human Research and Ethics Committee and all patients provided written informed consent before being enrolled in the study.

Basic demographic data was collected, together the time post-transplant and indication for bronchoscopy, acute rejection, immunosuppression regime, doses and levels, history of induction and CLAD according to updated guidelines (11). All patients were censored for CLAD and all-cause mortality in August 2020. Immunosuppression protocols consisting of a calcineurin inhibitor (largely tacrolimus), mycophenolate mofetil (MMF) and prednisolone have been uniform since the inception of this study, with basiliximab induction routine since April 2011.

### Bronchoscopy and Transbronchial Brushing

Bronchoscopy was performed either under conscious sedation with midazolam and fentanyl or general anesthesia with propofol, with lignocaine applied to the vocal cords and endobronchial tree. Bronchoalveolar lavage (BAL) was performed in accordance with the recent consensus statement for transplant recipients (12). Bronchial and bronchiolar airway brushings were then obtained on the same side as the BAL using standard cytology brushes as previously described (13). Briefly, the bronchoscope was wedged in a suitable lateral segment and a sheathed nylon cytology brush was passed down the working channel of the bronchoscope, unsheathed under radiologic guidance with the brush tip lying 2 to 3 cm from the pleural surface and gently agitated to collect bronchiolar samples. Trapped cells were tapped off into cooled 0.9% saline between brushes (n=4 per region). After the final brush, the tip was cut off and vortexed to remove adherent cells. Once the transbronchial brushing was performed, transbronchial biopsies were obtained and the lung apex then screened to exclude a pneumothorax as part of routine care. Bronchial brushes were obtained last, using a new brush, from a convenient large airway source distal to the airway anastomosis.

### Sample Preparation

Bronchial and bronchiolar airway samples were kept on ice and then transported to the laboratory where they were vortexed, and the brush tip removed. The samples were washed with RPMI before being stored at -80°C until batch analysis.

### Determination of Telomere Length by PCR

The relative telomere length in airway cells was determined by quantitative PCR based on the T/S ratio as previously described (14, 15). Briefly, genomic DNA was extracted from airway brushing samples using commercially available kits according to the manufacturer’s instructions (Qiagen). DNA was diluted to 2ng/µL and 20ng used in the PCR reaction. Multiplexed quantitative PCR was used to determine the telomere (T) and single copy gene (albumin, S) length using Sybr Green and a Viia7 (Applied Biosystems). Telomere length was compared to a standard curve made from serially diluted DNA from a mixture of 5 individuals. Positive and negative controls of known long and short (reference MCF7 cell line) telomere length were included with each run. Each sample was assayed in triplicate and the average Ct value used. The relative T/S ratio was calculated by dividing the sample T/S by the value of the reference T/S. The reported values are the average from four repeat experiments.

### Statistical Analysis

Results are presented as median (interquartile range, IQR) and statistical analysis was performed using STATA 14 (StataCorp, TX, USA). Group differences were assessed by Wilcoxon rank test, Mann-Whitney U test or Fisher’s exact test, as appropriate. Random intercept linear mixed models were used to longitudinally assess telomere length which included all available telomere lengths for an individual (median 2 samples/person, range 1-8). A random co-efficient model was also considered but not justified. Factors associated with telomere length were evaluated using the linear mixed models. The time from transplant to CLAD and all-cause mortality was modelled using Cox proportional hazard regression. Covariates included demographic factors, clinical factors such as time post-transplant and telomere length (limited to include those where the first measurement was within six months post-transplant). Covariates were tested and included in the model if p<0.1. The models retained covariates where predictors were statistically significant at α=0.05.

## Results

In total 231 bronchoscopies incorporating transbronchial brush and bronchial brush were performed in 120 patients. Full demographic data is shown in **Table 1**. Briefly, the median age at transplant was 50 years and 54% were male. The main indications for transplant were cystic fibrosis (37%) and COPD (35%), and 95% underwent a bilateral lung transplant. At the time of censoring, 43% and 35% of patients, respectively, had developed CLAD and had died. Median (IQR) follow up was 92.4 (77.8 – 110.5) months. A healthy control cohort consisting of individuals who had not undergone transplantation and who had no evidence of airways disease (n=10) was recruited with a median age of 64.4 (54.2 – 74.4) years and 20% male. The control cohort was significantly older (p=0.003) with more females (p=0.05).

**Table 1:** Cohort demographics.

	Cohort (n = 120)
Transplant age, median (IQR) years	49.7 (34.1 – 57.2)
Male sex, n (%)	65 (54)
Transplant type, n (%) Single Bilateral Heart & lung Heart, lung & liver	2 (2) 115 (95) 1 (1) 2 (2)
Pre-transplant diagnosis, n (%) CF COPD IPF other	44 (37) 42 (35) 19 (16) 15 (12)
Number of bronchoscopies/patient, median (IQR)	1 (1 – 2)
Time post-Tx (months) at first sample, median (IQR)	8.2 (2.8 – 31.8)
Donor age (years), median (IQR)	41 (28 – 52)
Any donor smoking history, n(%)	61 (58)
Donor pack-year smoking history, median (IQR)	13 (5 – 30)
Time of follow-up at census, median (IQR) months	92.4 (77.8 – 110.5)
CLAD at census, n (%)	51 (43)
Time of CLAD onset, median (IQR) months	46.5 (22.2 – 62.6)
Death, n (%)	42 (35)
Survival post-Tx (months), median (IQR)	61.0 (32.2 – 92.3)

### Telomere Length Is Conserved Between the Airways

**Figure 1:**
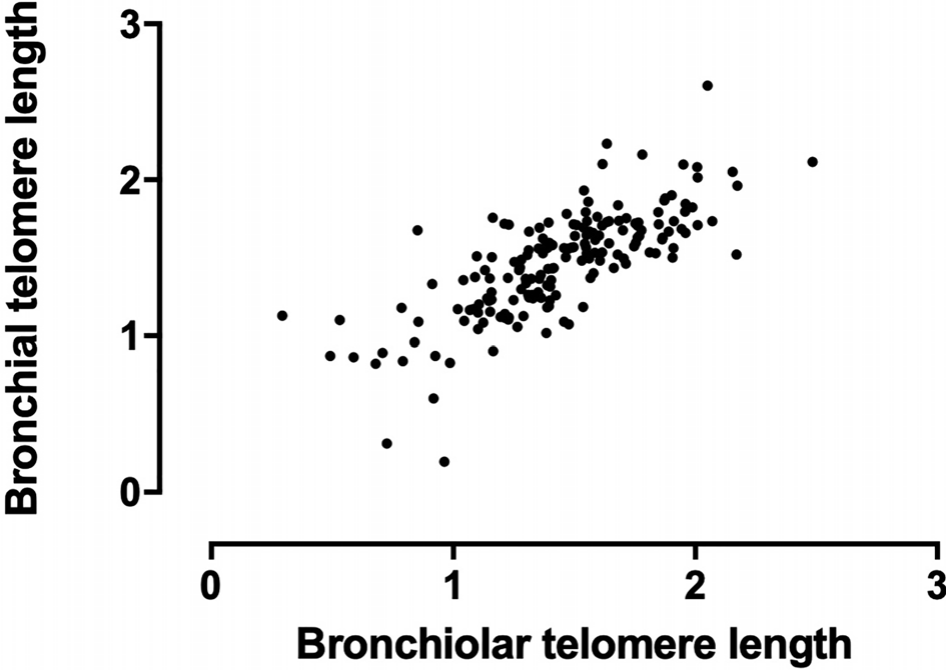
Correlation of bronchial and bronchiolar airway telomere length. Bronchiolar and bronchial airway telomere length are presented as a relative T/S ratio (arbitrary units).

Bronchoscopy samples were available from a median of one (IQR 1-2) bronchoscopy per patient with the first bronchoscopy occurring at 8.2 (2.8-31.8) months post-transplant. A second sample was available for 65 (28%), a third in 33 (14%) and a fourth or more in 13 (5%) of participants. Airway bronchiolar and bronchial telomere lengths were strongly correlated (r=0.78, p<0.001, **Figure 1**), confirming conservation of telomere length with airway branch generation.

### Airway Telomere Length Decreases With Age

**Figure 2:**
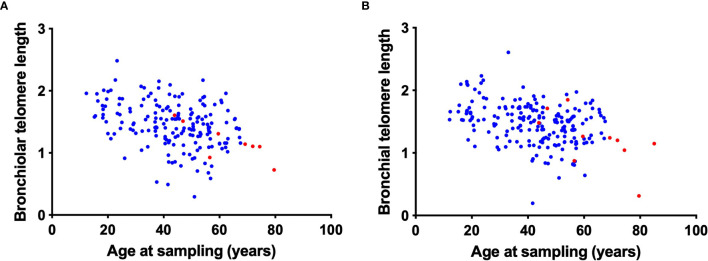
Correlation of bronchiolar and bronchial airway telomere length with donor age. Bronchiolar **(A)** and bronchial **(B)** airway telomere length (relative T/S ratio, arbitrary units) are plotted against the age at sampling for transplant recipients (blue). Age at sampling was calculated as the sum of donor age and time post-transplant at sampling. Control airway telomere length (red) are plotted against age at sampling.

We next investigated the relationship between airway telomere length and age in transplant recipients and controls. For transplant recipients we used the age of the allograft (donor age plus the time post-Tx). As expected, both the bronchiolar (r = -0.34, p<0.001) and bronchial (r = -0.31, p<0.001) telomere length decreased with age (**Figure 2**). Furthermore, the transplant airway telomere length was comparable to controls when adjusted for age. As expected, there was no relationship between recipient age and transplanted airway telomere length (data not shown). After adjustment for donor age and time post-transplant, donor sex was not associated with either bronchiolar or bronchial telomere length.

### Donor Age and Smoking History Are Associated With Shortened Airway Telomere Length

To understand what factors were associated with airway telomere length, regression models of telomere length from all bronchoscopies were developed. Factors investigated included demographics, donor age, time post-transplant, cytomegalovirus mismatch (donor seropositive, recipient seronegative), donor smoking pack year history and ischemic time. Neither the bronchiolar (**Table 2**) nor bronchial (**Table 3**) telomere length were associated with recipient demographics, time post-transplant, ischemic time or cytomegalovirus mismatch. However, shorter airway bronchiolar telomere length was associated with older donor age and shorter bronchial telomere length was independently associated with both older donor age and higher donor pack-year smoking history.

**Table 2:** Factors associated with bronchiolar telomere length (n = 120).

Univariable	β (95% CI)	p
Donor age (per 10 years)	-0.095 (-0.137- -0.053)	<0.001
Smoking history (per 10 pack years)	-0.067 (-0.122 - -0.012)	0.017
Time post-transplant (per 5 years)	0.030 (-0.074 - 0.135)	0.573
CMV mismatch	-0.073 (-0.234 – 0.870)	0.369
Ischaemic time (per hour)	0.007 (-0.0017 – 0.033)	0.554
Female sex	-0.033 (-0.156 – 0.090)	0.598
Pre-transplant diagnosis (compared to CF) COPD IPF other	-0.121 (-0.268 – 0.026) -0.145 (-0.331 – 0.040) -0.097 (-0.302 – 0.107)	0.107 0.125 0.350
Donor female sex	-0.045 (-0.178 – 0.085)	0.493
PGD grade (24 hours) compared to PGD0 PGD1 PGD2 PGD3	-0.066 (-0.228 – 0.095) -0.051 (-0.241 – 0.139) -0.047 (-0.255 – 0.151)	0.421 0.597 0.658
Final independent variable		
Donor age (per 10 years)	-0.095 (-0.137- -0.053)	<0.001

**Table 3:** Factors associated with bronchial telomere length (n = 120).

Univariable	β (95% CI)	p
Donor age (per 10 years)	-0.076 (-0.113 - -0.039)	<0.001
Smoking history (per 10 pack years)	-0.065 (-0.109 - -0.021)	0.004
Time post-transplant (per 5 years)	-0.021 (-0.101 0.059)	0.606
CMV mismatch	-0.059 (-0.196 – 0.078)	0.399
Ischaemic time (per hour)	0.005 (-0.019 – 0.029)	0.676
Female sex	0.021 (-0.090 – 0.132)	0.705
Pre-transplant diagnosis (compared to CF) COPD IPF other	-0.109 (-0.238 -0.019) -0.085 (-0.249 – 0.078) 0.013 (-0.164 – 0.191)	0.095 0.307 0.884
Donor female sex	0.012 (-0.104 – 0.128)	0.838
PGD grade (24 hours) compared to PGD0 PGD1 PGD2 PGD3	-0.009 (-0.156 – 0.138) -0.051 (-0.224 – 0.127) -0.027 (-0.223 – 0.169)	0.905 0.565 0.787
Multivariable		
Donor age (per 10 years)	-0.063 (-0.100 - -0.027)	0.001
Smoking history (per 10 pack years)	-0.044 (-0.087 - -0.002)	0.039

### Airway Telomere Length Is Not Associated With Time Post-Tx or CLAD/Survival

Finally, we investigated whether airway telomere length was associated with time to CLAD or all-cause mortality (**Table 4**). Given the cross-sectional nature of the study the analysis was restricted to only those whose airway telomere length was measured during the first six months post-transplant to approximate the starting telomere length (n=44). Neither the bronchiolar (HR 0.39 (0.06-2.3), p=0.30) nor the bronchial (HR 0.66 (0.2-1.7), p=0.39) airway telomere length were associated with the development of CLAD. Similarly, neither the bronchiolar (HR 0.92 (0.2-4.5), p=0.92) nor the bronchial (HR 0.47 (0.1-1.9), p=0.28) airway telomere length were associated with all-cause mortality. No associations between airway telomere length and outcome were observed when the analysis included the full cohort and all measured values were included in a time-dependent regression model (data not shown).

**Table 4:** Cox proportional hazards analyses for CLAD-free and overall survival limited to those with a telomere length measurement within 6 months of baseline (n = 44).

Univariable	CLAD-Free Survival		Overall Survival	
	HR (95% CI)	p	HR (95% CI)	p
Bronchiolar telomere length	0.39 (0.06 – 2.30)	0.300	0.921 (0.189 – 4.492)	0.920
Bronchial telomere length	0.66 (0.2 -1.7)	0.39	0.466 (0.115 – 1.885)	0.284
Female sex	1.216 (0.392 – 3.773)	0.7352	1.697 (0.631 – 4.566)	0.294
Tx age	0.997 (0.952 – 1.045)	0.918	1.003 (0.962 – 1.045)	0.890
Diagnosis (compared to CF) COPD IPF Other	1.959 (0.490 – 7.838) 1.140 (0.190 – 6.824) 1.230 (0.128 – 11.830)	0.342 0.886 0.858	1.078 (0.362 – 3.209) 0.246 (0.030 – 2.046) 1.139 (0.229 – 5.651)	0.893 0.194 0.874
Donor age	0.993 (0.955 – 1.031)	0.704	0.975 (0.943 – 1.008)	0.140
CMV mismatch (D+/R-)	1.813 (0.542 – 6.061)	0.334	1.197 (0.384 – 3.731)	0.756
Donor smoke	1.921 (0.520 – 7.100)	0.327	0.972 (0.344 – 2.745)	0.958

## Discussion

In this study we demonstrate that donor airway telomere length is conserved across the bronchial and bronchiolar airways and is strongly correlated with donor age and influenced by donor smoking history. However, no relationship was observed between donor airway telomere length and long-term post-lung transplant outcomes, namely CLAD and all-cause mortality. These data contrast with other studies which identified associations between shorter donor telomere length and CLAD, however the number of evaluable participants in this study was small, and there are notable between study differences in methodology.

Several prior studies have reported associations between donor airway telomere length and CLAD. Faust et al. demonstrated a robust association between donor PBMC or spleen cell telomere length and CLAD, as well as shorter early post-transplant (<90 days) endobronchial telomere length in a small sub-group of participants who developed CLAD compared to those who remained CLAD-free (7). Naikawadi et al. measured the telomere lengths of CLAD lung explants and found epithelial telomere length to be significantly shorter than in unused healthy donor lungs (10). This latter observation in itself does not necessarily implicate telomere shortening in the pathogenesis of CLAD, since, as the authors point out, it is unclear whether this association is a cause or a consequence of CLAD pathology. Put another way, the telomere shortening observed in CLAD lungs may simply be a biomarker of recurrent injury, reflective of the repetitive insults that cause CLAD. To further investigate the association, the authors constructed a murine model of club cell telomere shortening and demonstrated CLAD-like pathology in the transgenic mice (10). While both Faust et al. and Naikawadi et al. detected a difference between the early lung biopsy telomere lengths of those who did or did not subsequently develop CLAD, the sample sizes are small and it is not clear if the groups were matched for donor age or other factors (7, 10). In contrast, we were unable to demonstrate an association between early post-transplant airway telomere length and CLAD.

There may be several explanations for these contrasting observations. It is possible that the influence of donor telomere length relates to cells other than those present in the allograft airway. Faust et al. measured the telomere lengths of donor peripheral blood mononuclear cells and demonstrated that shorter donor telomere length in these cells was associated with an increased risk of CLAD or death with the greatest risk observed in young recipients of short telomere donors (7). In contrast, Courtwright et al. measured donor lymphocyte telomere length in 45 lung donors and found no association with recipient survival (8). However, median follow-up was only 2.1 years and the risk of CLAD was not assessed. Although it is not definitively known whether telomere length is conserved between peripheral blood and the lung, Saferali et al. (16) demonstrate a correlation in subjects with COPD and the study from Everaerts et al. (9) suggests compartmentalization of cellular telomere length in these locations. Other investigators have similarly noted differences in telomere lengths between peripheral blood and solid organs (17). We have demonstrated rapid replacement of donor-derived alveolar cells with recipient cells after transplantation (2), however we cannot exclude the possibility that short telomere passenger donor cells located outside the alveolar space and airway may persist to increase CLAD risk. Of course, another possibility for the discordant findings in these studies may be a lack of power to detect an increased risk of CLAD due to low numbers or insufficient follow-up.

In other transplant settings, donor telomere length has demonstrated importance. In hematopoietic cell transplant, short donor telomeres have been associated with worse survival (18) and late graft failure (19). The major difference with lung tissue is the high rate of turnover of bone marrow progenitor cells, predisposing to telomere exhaustion and senescence. By comparison, the turnover of lung epithelial cells is slow. The pathogenesis of CLAD is complex and not simply the result of a single molecular pathway, like telomere exhaustion and senescence. Both alloimmune and non-alloimmune pathways are implicated. Our results do not exclude telomere shortening as an insult, among others, that contributes to the pathogenesis of CLAD. Notably Naikawadi et al. demonstrated the presence of senescent cells in CLAD affected lung allografts, and their elegant transgenic mouse model was able to recapitulate CLAD-like pathology after induction of telomere shortening in club cells (10).

In comparison, recipient telomere length has consistently been shown to alter post-transplant outcomes, particularly in those with very short telomeres (20). A significant proportion of idiopathic pulmonary fibrosis is attributable to telomere related gene mutations (21–24). Patients with pulmonary fibrosis related to such mutations, with extreme telomere shortening, may be more prevalent in the transplant population, owing to their earlier age of onset and worse prognosis. Inferior post-transplant outcomes are noted in this group (20, 25–28). These patients appear more susceptible to the effects of immunosuppression with more frequent cytopenia (7, 20), an increased susceptibility to infective insults, in particular cytomegalovirus disease (20, 27) and extrapulmonary organ dysfunction (25). Reduced CLAD-free survival has been noted for this group of patients (20). These findings suggest that the effect of recipient telomere length is greatest in extrapulmonary organs, particularly the recipient immune system.

Our study has several limitations. The sample size of our study, in particular those with airway samples available from the first six months post-transplant is small. Furthermore, the narrow range of donor age in our cohort may have limited our ability to assess the very extremes of telomere length. However, our donor age range is similar to that of other cohorts and registries (1). Additionally, our follow-up is sufficiently long to capture most CLAD events. In comparison to the studies by Courtwright et al. (8) and Faust et al. (7), and the current demographics of transplant recipients internationally (1), our cohort had only a small number of pulmonary fibrosis recipients (16%). While this may be relevant to relationships between recipient telomere length and lung transplant outcomes, it is less likely to be relevant to donor airway telomere length, unless a double hit phenomenon exists. The increased risk of CLAD in short telomere recipients might perhaps relate to the shortened telomeres of the recipient cells that rapidly replace the donor alveolar space (2). These hypotheses require further exploration.

In summary, starting donor telomere length in the lung allograft airway is not a strong predictor of the future development of CLAD. This is fortunate since donor telomere length is not modifiable and is not feasibly measured in real-time during the process of organ donation. This data supports the continued acceptance of suitable older donor organs. Further study on the observed association between donor smoking history and airway telomere length and the contribution of telomere attrition to the pathogenesis of CLAD is warranted.

## Data Availability Statement

The raw data supporting the conclusions of this article will be made available by the authors, without undue reservation.

## Ethics Statement

The studies involving human participants were reviewed and approved by The Prince Charles Hospital Human Research and Ethics Committee. The patients/participants provided their written informed consent to participate in this study.

## Author Contributions

SY and DC developed the research concept. SY, MT, and LS performed experiments and analysed data. PH and DC recruited patients. SY performed statistics and produced figures. JM, SY, and DC interpreted data and drafted the manuscript. All authors contributed to the article and approved the submitted version.

## Funding

Funding was received from The Prince Charles Hospital Foundation.

## Conflict of Interest

The authors declare that the research was conducted in the absence of any commercial or financial relationships that could be construed as a potential conflict of interest.
